# The Dispersion and Hydration Improvement of Silica Fume in UHPC by Carboxylic Agents

**DOI:** 10.3390/ma17174253

**Published:** 2024-08-28

**Authors:** Taige Wu, Honghu Wang, Zhidan Rong

**Affiliations:** 1 School of Engineering, University of Birmingham, Birmingham B15 2TT, UK; gezhiwtg@163.com; 2 China Machinery International Engineering Design & Research Institute Co., Ltd.—East China Branch, Nanjing 210023, China; whh20052725@163.com; 3 School of Materials Science and Engineering, Southeast University, Nanjing 211189, China

**Keywords:** silica fume, polyacrylic acid, surface modification, hydration speed, mechanical strength, micro-structure

## Abstract

Silica fume (SF) is an essential component in ultra-high-performance concrete (UHPC) to compact the matrix, but the nucleus effect also causes rapid hydration, which results in high heat release and large shrinkage. In this paper, the carboxylic agents, including polyacrylic acid and polycarboxylate superplasticizer, were used to surface modify SF to adjust the activity to mitigate hydration at an early time and to promote continuous hydration for a long period. The surface and dispersion properties of modified SF (MSF), as well as the strength and pore structure of UHPC, were studied, and the stability of the modification was also investigated. The results demonstrated that, after treatment, the carboxylic groups were grafted on the SF surface, the dispersion of SF was improved due to the increased negative pentanal of the particle surface and the steric hindrance effect, the early hydration was delayed about 3–5 h, and the hydration heat release was also mitigated. The compressive strength of UHPC with MSF reached a maximum of 138.7 MPa at 3 days, which decreased about 3.7% more than the plain group, while flexural strength varied insignificantly. More pores and cracks were observed in the matrix with MSF, and the hydration degree was promoted with MSF addition. The grafted group on SF fell off under an alkali environment after 1 h.

## 1. Introduction

Concrete is the most popular and important global construction material. The requirement of concrete may exceed 10 billion tons per year with the rapid development of society and the economy [1]. However, with the requirements of low-carbon development and natural resources protection, the development of green concrete is urgent [2,3]. Ultra-high-performance concrete (UHPC) is a new generation of concrete materials that has attracted lots of attentions in recent decades. The strength of UHPC ranges from 100–800 MPa, much stronger than normal concrete (NC), and the impermeability and corrosion resistance of UHPC are tens of times higher than NC [4,5,6,7,8]. It can be used not only in thin-wall, long-span structures and other specific structures to reduce the material costs but also in severe environments like marine or salt-lake conditions to increase the service life [9,10,11,12]. UHPC shows great potential in low-carbon development in the full cycle of construction life [13,14].

To achieve such outstanding properties, the design of UHPC follows the dense packing theory, and silica fume (SF) is an important component that supplies the filling effect, nucleation effect, and pozzolanic effect [15,16]. Zhang [17] prepared seawater UHPC using SF and found that with a dosage of 10%, the compressive strength improved, especially at a later time Xi [18] proved that SF accelerates the early hydration of UHPC using the seeding effect and preferential adsorption and enhances the strength using the pozzolanic reaction. However, the promotion of SF in early hydration also caused some disadvantages, such as high hydration temperature and autogenous shrinkage [19,20]. Li [21] demonstrated that the heat release of UHPC increased with the dosage of SF using simulation and experimentation. Therefore, reducing the hydration rate at an early time but allowing it to retain the strength and impermeability of UHPC is an available method to widely develop this material. Plenty of works were performed to replace SF with other mineral admixtures to save material costs and reduce the autogenous shrinkage [22,23,24,25,26]. Xing [27] replaced SF with inert mineral powders to prepare UHPC, and the strength declined from 208 MPa to 131 MPa, while the pore size also shifted from 10 nm to 100 nm. Jin [28] used submicron autoclaved aerated concrete waste to replace SF with a different ratio and found that early hydration was promoted with more submicron substitution. The strengths at 7 and 28 days also increased. Based on our survey, the substitution of SF with different ratios all caused a reduction in strength, and research that focuses on the effects of SF on hydration heat were rarely reported.

Surface modification on components is an available method to adjust the hydration of concrete [29,30,31,32,33,34], and a polycarboxylate acid agent is frequently selected, due to its compatibility with the cement system. Huang [35] used a polycarboxylate ether-based (PCE) superplasticizer to modify nano-silica to form a core-shell structure and improve the dispersion of nano-silica particles. Skripkiunas [36] studied the effect of multi-wall carbon fibers on self-consolidating concrete (SCC), and the PCE-modified carbon fibers decreased the plastic viscosity from 29.6% to 9.9%; the yield stress decreased to 0 Pa. Besides that, researchers used PCE to improve the dispersion of nano-sized particles. Since SF partly reached the nano-scale, agglomeration existd to weaken the filling and nano-nucleation effect. Mao [37] studied the dispersion properties of SF and proved that the dispersion of SF was unaffected by the superplasticizer, and the aerodynamic dispersion method significantly reduced silica fume agglomeration. Gu [38,39] grafted polycarboxylic acid on the SF surface to prepare SF@ PCE core-shell structure particles and proved that the modification significantly improved the SF dispersion, accelerated cement hydration, and affected the transformation of AFt to AFm. Fen [40] used a silane and polycarboxylic coupled agent to modify SF and delay the early hydration time. Ma [41] used silane and polyacrylic acid to treated SF, and the modified SF in UHPC decreased the total hydration heat by about 25.7% and increased the hydration degree by 16.2%. However, the research above were all focused on the physical dispersion of SF particles, and the effects on cement hydration are still unclear.

In this paper, a new surface modification method that affects the dispersion was investigated, and the hydration activity of SF was designed to work in a different stage. At an early time, the cement hydration was dominated, and the SF particle inhibited the pozzolanic reaction and cement hydration promotion by blocking the active surface. However, this block would fall off in the alkali solution caused by cement hydration, exposing the active surface again to increase the hydration degree of UHPC for the long term.

## 2. Materials and Methods

### 2.1. Raw Materials

**Figure 1:**
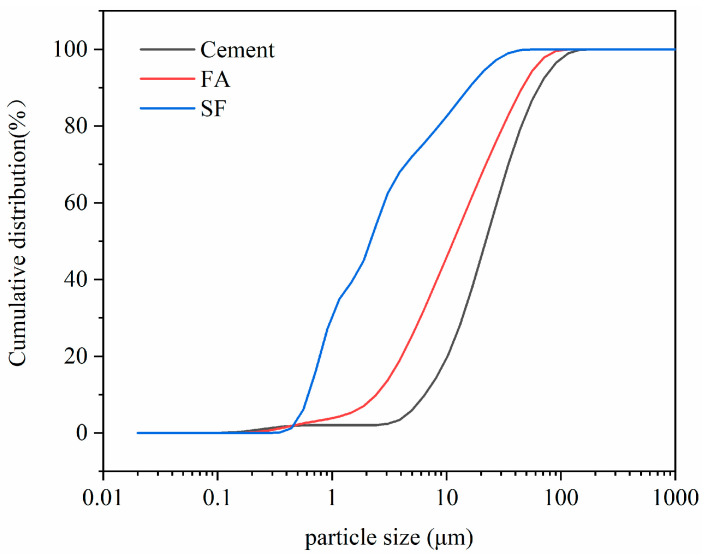
Cumulative size distribution curves for raw material particles.

**Figure 2:**
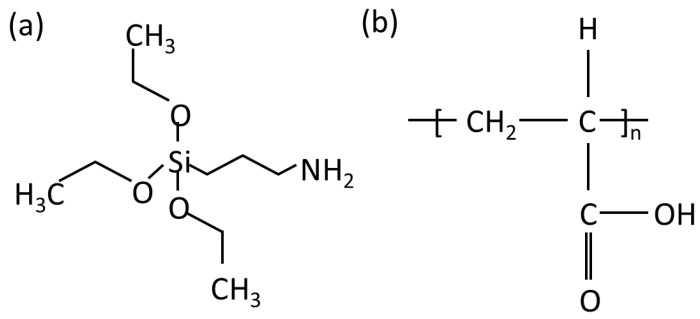
Molecule structures of (**a**) KH-550 and (**b**) PAA.

**Figure 3:**
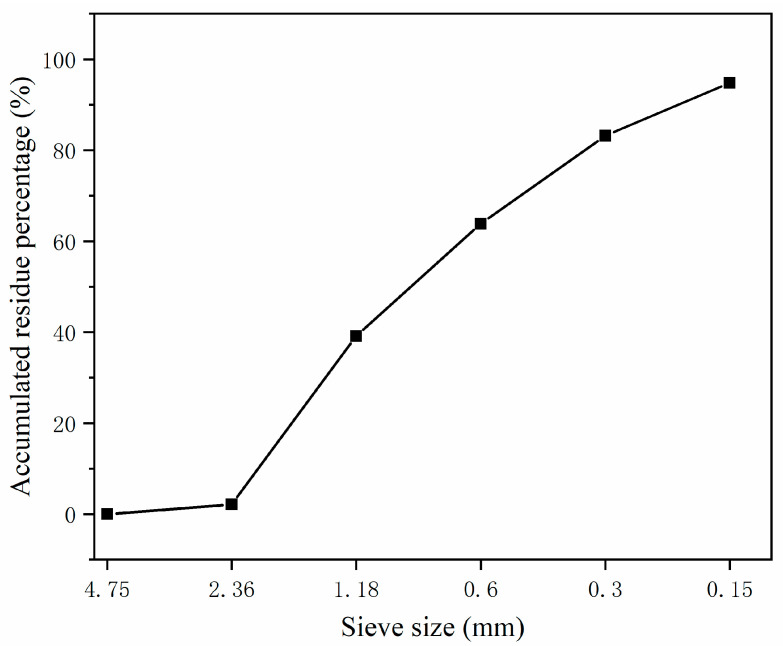
The sieve curve of sand.

Portland cement (PC) from Conch Cement Co., LTD of Chizhou, China, fly ash (FA), and silica fume (SF) which were by-products from local factories of Anhui were used as binder materials in the experiment. The chemical composites of binders and the physical index of SF are listed in Table 1. The particle size distribution of the binders is presented in Figure 1. The chemical agents, including γ-aminopropyl triethoxysilane (KH-550), polyacrylic acid (PAA), and polycarboxylate water reducer (WR), were used to modify SF. The indices of the agents are shown in Table 2, and the molecule structures are revealed in Figure 2; the structure of WR was more complex. River sand with a fineness modulus of 2.81 and with a size smaller than 2.36 mm was chosen as the aggregate. The sieve curve of sand is shown in Figure 3.

### 2.2. Modification and Mixture Process

**Figure 4:**
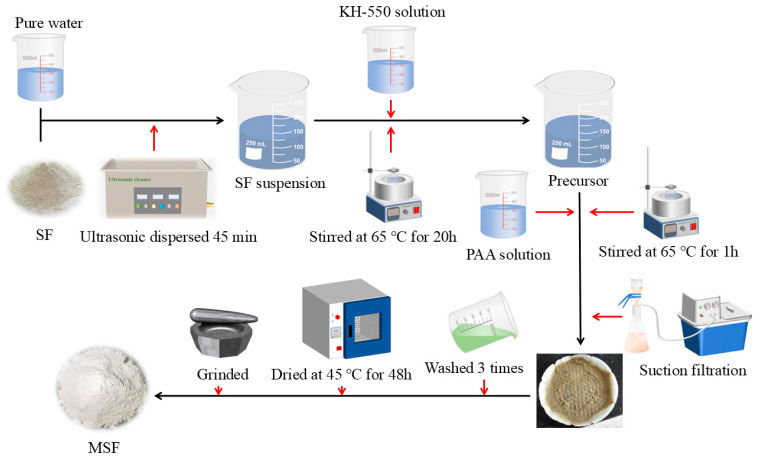
The sketched diagram of the SF modification process.

For SF surface modification, based on our previous research [41], 20 g of SF was first ultrasonically distributed into 600 mL of ultrapure water for 45 min; then, 100 mL of KH-550 solution with a concentration of 3% was added into the SF solution and stirred at 65 °C for 20 h. After that, a specific dosage of the modification agent was first diluted with 100 mL of ultrapure water and then added into the SF mixture and mixed for another 1 h at 65 °C. The treated SF was filtered and washed with ultrapure water and anhydrous ethanol several times; then, it was dried at 45 °C under vacuum for 48 h and ground into fine powder for use. The modified SF was named MSF. The dosages of the modification agent are presented in Table 3, and the treatment process is shown in Figure 4.

To prepare the cementitious mortar, the binders of PC, FA, MSF, and sand were dry mixed for 1 min. Then, water and PCE were added and continuously mixed for 5 min until mortar formed. The mortar was casted into a mold with a size of 40 mm × 40 mm × 160 mm, placed under the standard condition (20 °C ± 5 °C, RH > 95%) for 1.5 days, and then demolded and stream-curried at 60 °C for 3 days [42]. The mixed proportion of plain mortar is listed in Table 4, and the SF was replaced by a different modified SF with the same dosage.

### 2.3. Method

The particle size distribution of SF with different treated agents was tested using the laser particle size analyzer (Bettersize, Dandong, China). About 5 g of powder was first dispersed in 100 mL of ultra-pure water and then dropped into the test container for testing.

The surface properties of raw and modified SF were evaluated by Zeta potential and the Fourier-transform infrared spectroscopy (FT-IR) method. For the Zeta potential test, the sample particles were first dispersed in pure water, and then the solution was tested 3 times to obtain an average. For the FTIR test, about 10 g of the sample was first ground with KBr to achieve a full mix and then pressed into a piece for testing. Also, to evaluate the stability of the modification agent, the MSF samples were immersed in water or a Ca(OH)_2_-saturated solution for different periods of time. Then, the sample was dried at 40 °C for 48 h and tested by FITR.

The powder morphology was observed by scanning electronic microscopy (SEM) (Regulus, Tokyo, Japan). For SF or MSF particles, the sample was dispersed in ethanol and dropped on the specimen stage for testing. For UHPC samples, an appropriate hardened matrix was chosen and sprayed gold for 1 min before testing.

The hydration process of cement with MSF was monitored using the isothermal conduction calorimetry method. The mix proportion of the sample was the same as UHPC, excluding sand, and about 20 g of the total sample was set in the exam channel once the cement was mixed with water. The exam was performed at 20 °C for 72 h.

The compressive and flexural strengths of mortar were tested followed the Chinese standard “Test method of cement mortar strength (ISO method)” [43], and the hydration products were also analyzed by X-ray diffraction (XRD) (Rigaku, Tokyo, Japan). For XRD quantitative analysis, the sample was ground into powder with particles smaller than 80 μm. Analytically pure α-Al2O3 and the sample powders were mixed uniformly in a mass proportion of 1:9 for the quantitative analysis. The scanning pace and the scanning angular range were 0.30 s/step and 5°–90°, respectively.

The porosities of UHPC samples were also tested using the mercury intrusion porosimetry method (MIP).

## 3. Results and Discussion

### 3.1. The Properties of Modified SF

**Figure 5:**
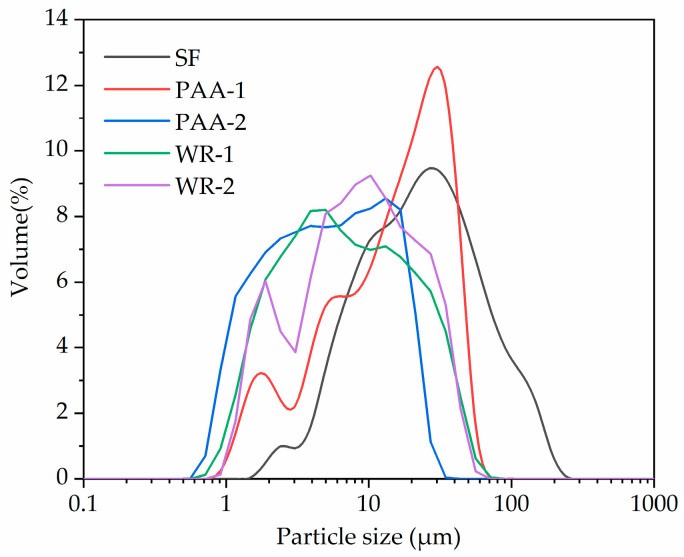
Particle size distributions of raw SF and MSF with different methods.

Since the size of SF reached the nano-scale, it was possible for the agglomeration to happen in raw particles. The particle size distributions of SF and MSF treated with different agents and dosages are displayed in Figure 5.

It can be seen from Figure 5 that the fraction of small-size particles in all modified SFs was larger than that in raw materials. This proved that the modification process broke the initial clusters and improved the dispersion. Still, a greater PAA dosage led to a better dispersion, especially for the particle ranged less than 2 μm. Additionally, the efficiency of WR was higher than PAA with a low dosage but did not significantly change with different WR additions.

**Figure 6:**
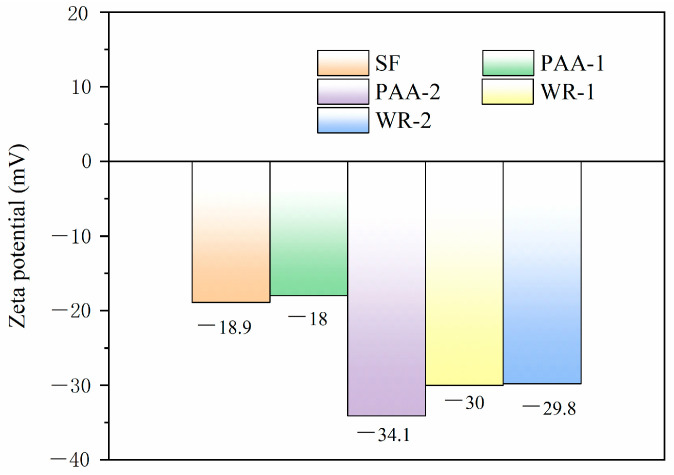
The Zeta potentials of SF with modifications.

**Figure 7:**
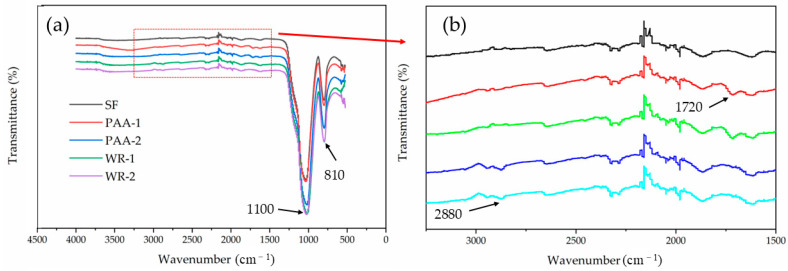
The FTIRs of different SF samples (**a**) and the enlarged particle view (**b**).

The surface pentanal and the functional group are illustrated in Figure 6 and Figure 7. From Figure 6, it can be seen that the pentanal of SF modified with 8 g of PAA was similar to that of the raw materials, which was nearly −18 mV. However, once the dosage of PAA increased to 12 g, the negative pentanal increased to −34.1 mV. Also, the surface pentanal of the sample modified by different dosages of WR was approximately the same, about −30 mV.

According to the electrostatic repulsion theory, two particles with higher surface pentanals would suffer a greater repulsion force. The higher negative pentanal of the modified SF was caused by the carboxyl from the branched chain of modification agents. Based on the results of Figure 4, the 12 g PAA worked with the most efficiency on the surface modification and caused the greatest repulsion force between SF particles. Therefore, sample PAA-1 exhibited the best distribution. The results were also in accordance with the particle size distributions above.

In Figure 7, the large peaks at 1100 cm^−1^ and 810 cm^−1^ were the asymmetric and symmetric stretching vibrations of Si-O-Si [41]. After integration, the area of the peak at 1100 cm^−1^ varied, with SF > PAA-1 > WR-1≈WR-2 > PAA-2. The lower peak area showed that a smaller amount of Si-O-Si was detected but was blocked with the modification agent.

In Figure 7b, the peak around 2880 cm^−1^, which only appeared in WR-1 and WR-2, was attributed to the symmetric stretching vibrations of -CH_2_ from polycarboxylic acid, and the band at 1720 cm^−1^ was attributed to the stretching vibration of the carbonyl group from PAA [38,41].

Figure 6 and Figure 7 prove that PAA and WR were grafted onto the SF surface after treatment, respectively. The coated area ratio of PAA-1 was the largest. The coated agent induced electrostatic repulsion and the steric hindrance effect, which helped with particle dispersion.

**Figure 8:**
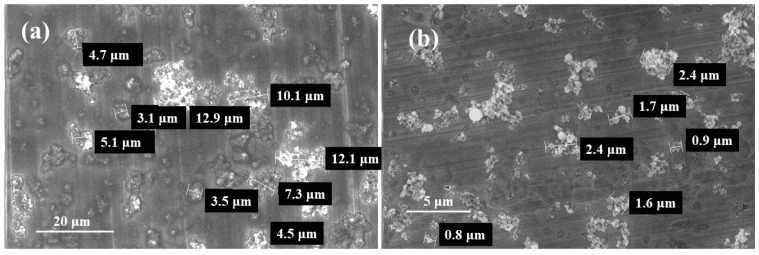
The SEM images of samples SF (**a**) and PAA-2 (**b**).

The morphologies of the samples are shown in Figure 8, and sample PAA-2 was taken as the example. Although the individual SF particles were rarely observed, after treatment, the size of the SF cluster significantly decreased from tens of micrometers to less than 3 μm. The particle dispersion was improved by PAA modification.

### 3.2. The Effect on UHPC

**Figure 9:**
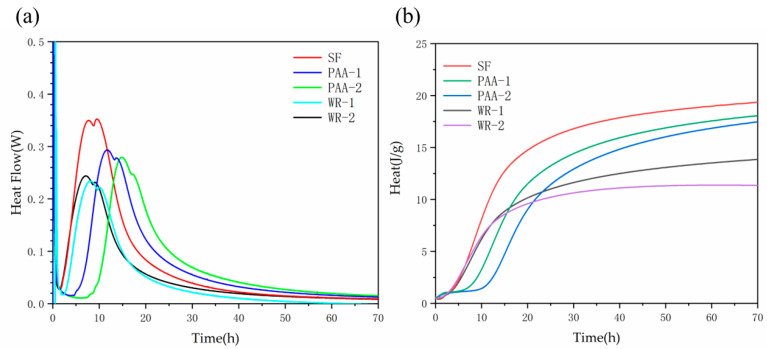
The heat flow (**a**) and heat (**b**) for paste with differently treated SFs.

The hydration process of UHPC paste with different treated SFs are shown in Figure 9. With the plain SF, the acceleration period began the earliest, with the greatest intensity of the heat flow peak and the highest heat release for the whole process. After it was modified with PAA, the hydration process was delayed about 3–5 h, and this effect was more significant with a higher PAA dosage; the total hydration heat was also less than that of plain SF. However, the decline rate of heat flow after the peak was also slower than that of plain SF, which indicated a stronger hydration for a longer period. Still, after WR treatment, the heat flow peak appeared later within 1 h, and the intensity was only half of the plain one. The dosage of WR was only slightly affected the hydration of SF, and the heat release after a long period was approximately the same as that of plain SF.

**Figure 10:**
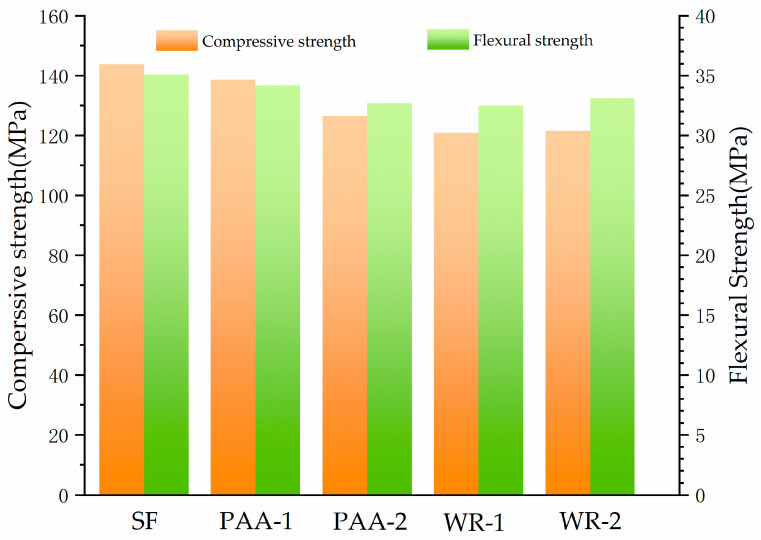
The compressive and flexural strengths of UHPC.

The compressive and flexural strengths of UHPC with different SFs were also tested and presented in Figure 10. The compressive and flexural strengths of UHPC reached 143.8 and 35.1 MPa, respectively. After modification, the compressive strengths for all samples dropped slightly. The decrease ratios of PAA-1 and PAA-2 were 3.7% and 12%, while the drop for that with the WR-modified SF was about 16.6%. The flexural strength also varied insignificantly. Based on this data, the PAA group works better than the WR group for maintaining strength.

Since the hydration speed and the heat release of modified SF all decreased at an early time, the reaction intensity of cement was restrained. Therefore, the strength of UHPC with a modified SF declined. However, from the results in Figure 10, the drop in strength was very limited.

**Figure 11:**
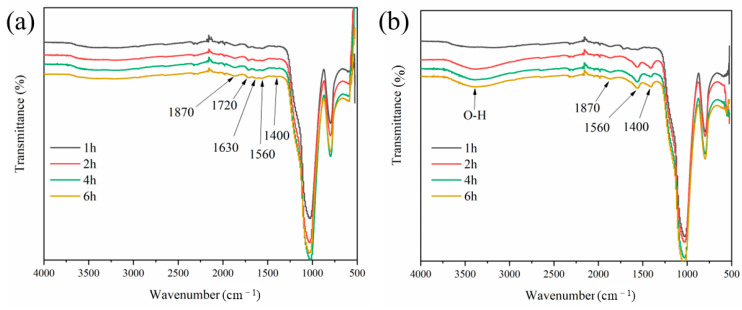
The MSF immersed in water (**a**) and Ca(OH)_2_-saturated solution (**b**) for different lengths of time.

To understand well the effect of PAA on cement hydration, the functional group of MSF was immersed in water and Ca(OH)_2_-saturated solution for different lengths of time, and the results are presented in Figure 11.

In Figure 11a, the small absorptions at 1720 cm^−1^ and 1630 cm^−1^ corresponded to the stretching and bending vibration of the carbonyl group from PAA, while 1560 cm^−1^ and 1400 cm^−1^ were attributed to the bending vibrations of N-H and C-H, which were from KH-550 [41]. Once the PAA-modified SF was immersed in water, the grafted functional groups were not changed, and the curves were approximate with the time. However, in Figure 11b, it was found that the groups corresponded to 1720 cm^−1^ and 1630 cm^−1^ from PAA and were detected after 1 h but disappeared after 2 h or longer, leaving groups mostly from KH-550. These results indicated that the carboxylic branch chains grafted on the SF surface could stably exist in water but would fall off under alkali conditions.

**Figure 12:**
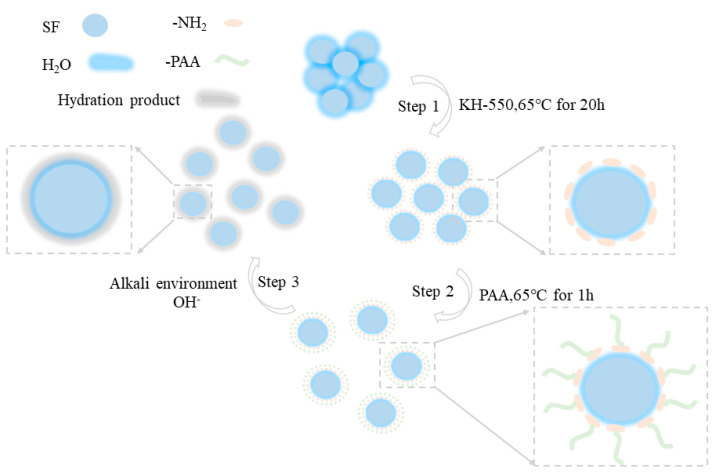
The sketched process of the status of PAA-modified SF.

In this case, when the modified SF was used in UHPC, because of the covered function group, the hydration activity was restrained, but the dispersion status improved. The SF particles were only used as filler. With the cement hydration and with enough alkali generated in the environment, the grafted groups fell off, and the active surface of SF was again exposed to work as the nucleus for cement hydration and to promote the strength development for the long term. The sketched process is illustrated in Figure 12.

**Figure 13:**
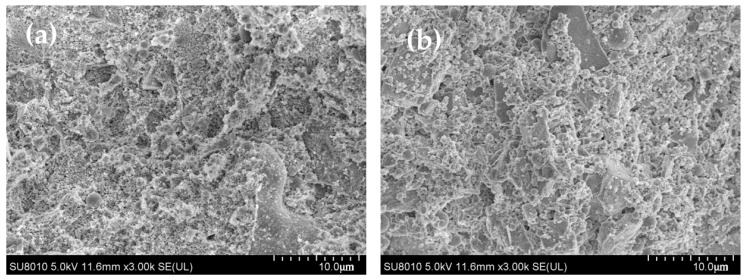
The morphologies of UHPC with plain SF (**a**) and PAA-modified MSF (**b**).

The micro-morphology of UHPC is shown in Figure 13. In plain SF, the matrix was more compacted, due to the rapid early hydration. With PAA-modified MSF, the matrix contained more pores, cracks, and rodlike products, which are supposed to be where AFt is found. This may be caused by the low hydration speed.

**Figure 14:**
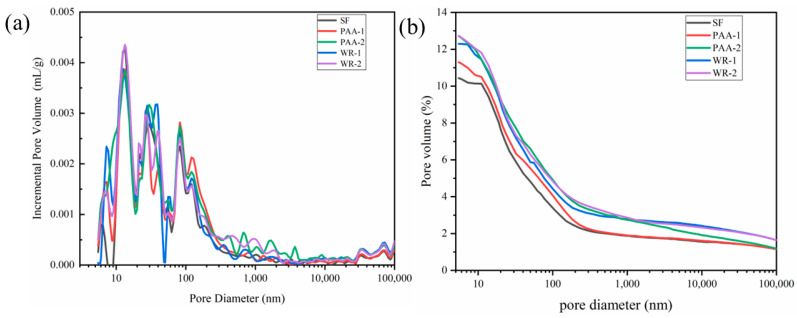
The pore size distributions (**a**) and total porosities (**b**)of UHPC with different MSFs.

The porosities of UHPC with different kinds of MSF were tested by MIP and are shown in Figure 14. As Figure 14a shows, the sample of PAA-1 formed more pores that ranged from 7–100 nm, which were classified as gel pores. This increase in gel pores also proved that more cement was hydrated in PAA. However, for WR-1 and WR-2, more pores were detected with larger sizes than 1 μm. From the curves of total porosity in Figure 14b, it was also found that although SF showed the lowest porosity, the increment of PAA-1 was focused on less than 200 nm, but macro-pores contributed more to other samples.

**Figure 15:**
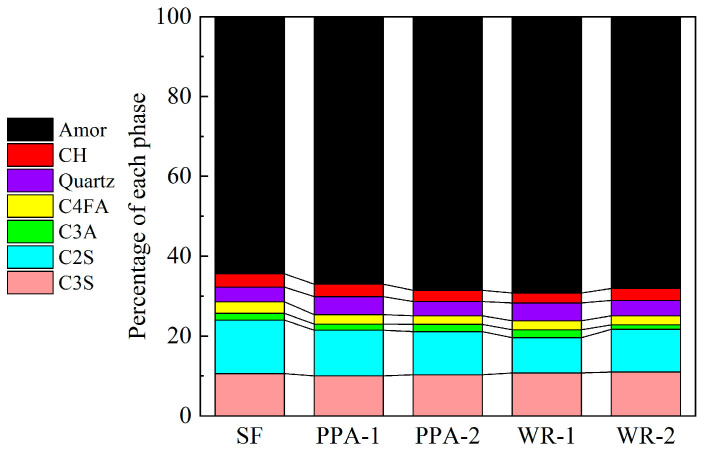
The contents of hydration products for UHPC.

The contents of the main components obtained from the XRD results are shown in Figure 15. The main crystalline phases identified in the composites were C_3_S, C_2_S, C_3_A, C_4_AF, calcium hydroxide, ettringite, alumina, and calcium carbonate. In addition, the hydration degree of HPCC was further calculated using the relative content of the four main mineral phases, C_3_S, C_2_S, C_3_A, and C_4_AF, in cement [44,45]. The calculation formula is as follows [46,47]:

(1) $$OH=1-\frac{w_{C_{3}S}\left(t\right)+w_{C_{2}S}\left(t\right)+w_{C_{3}A}\left(t\right)+w_{C_{4}AF}\left(t\right)}{w_{C_{3}S}\left(t_{0}\right)+w_{C_{2}S}\left(t_{0}\right)+w_{C_{3}A}\left(t_{0}\right)+w_{C_{4}AF}\left(t_{0}\right)}$$

where w_i_(t) represents the residual mass fraction of the different minerals at the time of t, while w_i_(t_0_) represents the initial mass fraction of the different minerals.

**Figure 16:**
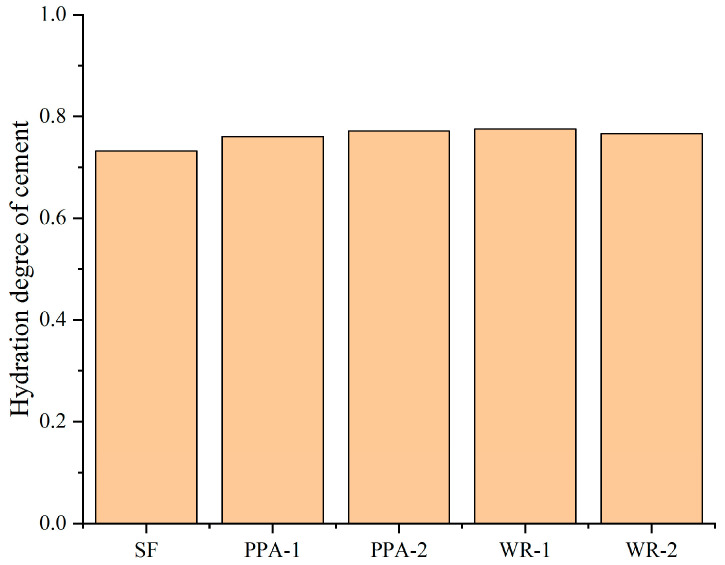
Hydration degrees of UHPC.

The calculated hydration degree of UHPC is shown in Figure 16. The hydration degree of UHPC was improved by the addition of MSF, which is consistent with the previous heat evolution test results.

## 4. Conclusions

In this paper, SF was modified with PAAs and WRs of different dosages to adjust the hydration speed of UHPC. The surface properties, dispersion, and the strength of UHPC with MSF were investigated, and the micro-structure properties were also studied to reveal the changes in strength. The results are concluded below:

(1) After modification treatment, the function groups of PAA and WR were successfully grafted onto the SF surface. The grafted group increased the negative potential, which was stronger than the electrostatic repulsion between particles, and this repulsion, coupled with the steric hindrance effect, improved the dispersion of SF after treatment; the PAA with a dosage of 12 g showed the greatest dispersion efficiency.
(2) The acceleration period of UHPC was delayed with MSF, and the hydration heat also declined. However, with PAA modification, the increased rate of heat at a later period was higher than plain SF.
(3) The strengths of UHPC slightly dropped once MSF was used, and the maximum decreased ratio of the compressive strength reached 15.9%; however, the value still exceeded 120 MPa, and the change in flexural strength was negligible.
(4) The porosities of UHPC with MSF were all greater than the plain group. However, in PAA-1, more gel pores were generated, due to the higher hydration degree, while in other samples, especially the WR-treated sample, more macro-pores were generated.
(5) The grafted functional groups from PAA on the SF surface were stable in water, but they fell under alkali conditions after 1 h. This phenomenon proved that the MSF worked only as filler at early hydration and promoted hydration as the nucleus after a long period.

However, the hydration products evolution and the mechanism of MSF on cement hydration still need further investigation.

## Figures and Tables

**Table 1:** Physical and chemical properties of binders (wt%).

Chemical	PC	FA	SF
SiO_2_	18.80	50.66	91.83
Al_2_O_3_	4.88	30.83	0.48
CaO	66.13	4.00	0.29
Na_2_O	0.15	0.90	0.11
MgO	1.29	0.92	0.33
Fe_2_O_3_	3.66	6.07	0.40
K_2_O	0.92	2.85	0.04
TiO_2_	0.26	1.16	0.03
MnO	0.04	0.54	0.01
SO_3_	3.50	1.68	0.97
L.O.I	3.54	2.75	1.20
Specific surface area (m^2^·g^−1^)	1.611	-	29.9
Specific gravity	2.4	-	2.3

**Table 2:** The physical indices of chemical agents.

Agent	Molecular Weight	Specific Gravity	Concentration	Color	Water Solubility
PAA	~3000	1.23	51.4%	colorless	soluble
KH-550	221.37	0.95	98.2%	colorless	soluble

**Table 3:** The dosage of the modification agent in the experiment (g).

Group	Agent	Dosage
PAA-1	PAA	8
PAA-2		12
WR-1	WR	8
WR-2		12

**Table 4:** The mix proportion of plain mortar (g).

Group	PC	SF	FA	Sand	Water	WR
Plain	600	100	300	1000	160	16

## Data Availability

The raw data supporting the conclusions of this article will be made available by the authors on request.
